# Reclassification of Clinical Familial Hypercholesterolemia Diagnoses After Adjustment of LDL-C for Lipoprotein(a) Cholesterol: A Systematic Review and Meta-Analysis

**DOI:** 10.3390/jcm15218614

**Published:** 2026-11-05

**Authors:** Grzegorz Procyk, Adam Sukiennik, Jakub Michal Zimodro

**Affiliations:** First Department of Cardiology, Medical University of Warsaw, 02-097 Warsaw, Poland; grzegorz.procyk@wum.edu.pl (G.P.); s088458@student.wum.edu.pl (A.S.)

**Keywords:** familial hypercholesterolemia, lipoprotein(a), reclassification, LDL-C

## Abstract

**Background/Objectives**: Low-density lipoprotein cholesterol (LDL-C) measurements include cholesterol carried by lipoprotein(a) [Lp(a)], which may influence the clinical classification of familial hypercholesterolemia (FH). We aimed to systematically review the available evidence and estimate the pooled proportion of individuals reclassified to a lower FH diagnostic category after correction of LDL-C for estimated Lp(a)-cholesterol. **Methods**: Embase, PubMed, and Scopus were searched from inception through 9 October 2026. We included observational studies with cross-sectional analyses of adults aged ≥18 years evaluated for suspected FH using the Dutch Lipid Clinic Network (DLCN) criteria before and after LDL-C correction for Lp(a)-cholesterol. Lp(a)-cholesterol was estimated as 30% of Lp(a) mass. The primary endpoint was reclassification to a lower DLCN diagnostic category among participants with possible, probable, or definite FH at baseline. Random-effects models were used to estimate pooled proportions. Risk of bias was assessed using the JBI critical appraisal tool for cross-sectional studies. **Results**: Four studies comprising 2970 individuals were included. The primary analysis included 2793 participants with possible, probable, or definite FH at baseline. After LDL-C correction for estimated Lp(a)-cholesterol, 19.8% (95% CI, 15.9–24.5) were reclassified to a lower DLCN diagnostic category. **Conclusions**: Correction of LDL-C for estimated Lp(a)-cholesterol resulted in downward DLCN reclassification in approximately one in five adults with at least possible FH from selected referral populations. The clinical utility of this adjustment, including its consequences for genetic testing and missed FH diagnoses, needs to be investigated.

## 1. Introduction

Familial hypercholesterolemia (FH) is a genetic disorder that presents with high to extremely high levels of low-density lipoprotein cholesterol (LDL-C), which translate into substantially increased atherosclerotic cardiovascular risk [1,2]. The diagnosis can be confirmed by identifying a pathogenic variant in an FH-associated gene through genetic testing. A clinical diagnosis or suspicion of FH can also be established using clinical diagnostic criteria. The Dutch Lipid Clinic Network (DLCN) score is one of the most commonly used tools [3]. This scoring system includes the assessment of LDL-C levels. However, measured LDL-C includes cholesterol carried by lipoprotein(a) [Lp(a)], which may affect diagnostic classification, particularly in individuals with elevated Lp(a) [4,5]. Several studies have reported reclassification rates after adjusting LDL-C for its estimated Lp(a)-cholesterol component [6,7,8,9]; however, to our knowledge, the proportion of patients undergoing diagnostic reclassification has not been systematically quantified through meta-analysis. We therefore aimed to systematically review the available evidence and estimate the pooled proportion of individuals whose clinical FH classification changes after adjustment of LDL-C for Lp(a)-cholesterol. We selected the DLCN criteria because they are widely used and distinguish four diagnostic categories, allowing changes in clinical FH classification after LDL-C adjustment to be quantified.

## 2. Materials and Methods

### 2.1. Eligibility Criteria

This systematic review and meta-analysis adhered to the PRISMA 2020 guidelines (Supplementary Material, Table S1) and was registered in PROSPERO under registration number CRD420261534815. We included observational studies with cross-sectional analyses that met all the following: (1) enrolled adults aged ≥18 years undergoing clinical assessment for suspected FH irrespective of genetic testing status; (2) assessed clinical FH classification both before and after adjustment of LDL-C for the estimated cholesterol content of Lp(a) and reported the proportion of participants whose FH classification changed to a lower diagnostic category; and (3) were published in English. Book chapters, case reports, conference abstracts, editorials, guidelines, letters to the editor, preprints, protocols, and reviews were excluded from the review.

### 2.2. Information Sources and Search Strategy

We searched three databases: Embase, PubMed, and Scopus from inception through 9 October 2026. The search strategy for each database is provided in the Supplementary Material (Method S1).

### 2.3. Selection Process

The study selection process was performed by two independent reviewers (G.P. and A.S.) using EndNote reference management software (version 21.5). First, the EndNote duplicate detection tool was used to identify duplicate records. Next, the reviewers independently screened all records to identify any remaining duplicates. Subsequently, both reviewers assessed eligibility based on titles and abstracts. Full-text articles were then evaluated for records considered potentially eligible. Any discrepancies were discussed and resolved by consensus.

### 2.4. Data Collection Process

Data from each eligible study were extracted independently by G.P. and A.S. and entered into a Microsoft Excel spreadsheet. Any discrepancies were resolved through discussion and consensus. Study authors were not contacted to obtain additional information.

### 2.5. Data Items

We collected data for the following: (a) proportion of participants whose clinical FH classification changed to a lower diagnostic category after adjustment of LDL-C for its estimated Lp(a)-cholesterol content; (b) the proportion of participants reclassified from possible to unlikely FH after LDL-C adjustment; (c) the absolute change in LDL-C concentration and clinical FH diagnostic score after adjustment; (d) changes in diagnostic performance for genetically confirmed FH, including mutation carriers newly classified as unlikely FH, sensitivity, specificity and area under the receiver operating characteristic curve (AUC), where reported. We also collected the number of individuals in each clinical FH classification group at baseline. For each study, we also collected data on baseline participant characteristics, including age (years), female sex (%), and lipid-lowering treatment use (%). In addition, the following study-level data were collected: total sample size, country of origin, and enrollment period.

### 2.6. Study Risk of Bias Assessment

The risk of bias was assessed independently by G.P. and A.S. using the JBI critical appraisal tool for cross-sectional studies [10]. Any discrepancies were resolved through consensus.

### 2.7. Effect Measures

Meta-analyses were restricted to results obtained using the DLCN criteria and LDL-C correction for estimated Lp(a)-cholesterol, calculated as 30% of Lp(a) mass. As described above, the DLCN criteria were chosen because they are widely used and distinguish four diagnostic categories, allowing changes in clinical FH classification after LDL-C adjustment to be quantified. Pooled proportions were reported with 95% confidence intervals (95% CIs) and 95% prediction intervals.

### 2.8. Statistical Analysis

For each outcome, all eligible studies providing the required data were included in the synthesis. For the primary and secondary endpoints, pooled proportions were estimated using a random-effects generalized linear mixed model (GLMM) with a binomial likelihood and logit link. Between-study variance (tau^2^) was estimated using the maximum likelihood (ML) method. The 95% prediction intervals were calculated using the Veroniki method. Heterogeneity was assessed using Cochran’s Q test and the I^2^ statistic. Results were presented as forest plots. Leave-one-out sensitivity analyses were performed for both pooled endpoints. Other outcomes were summarized descriptively. All statistical analyses were performed using R, version 4.5.2 (R Foundation for Statistical Computing). An AI-based language model (ChatGPT; GPT-6 Astra; OpenAI) was used to assist with conceptualization, data validation, development of statistical analysis code, and minor language editing of the manuscript; the authors remain fully responsible for all content.

### 2.9. Reporting Bias Assessment

Publication bias was not formally assessed and funnel plots were not generated due to the small number of included studies (n = 4).

### 2.10. Certainty Assessment

The certainty of the evidence was assessed for the primary and secondary endpoints. Five GRADE domains were considered: risk of bias, inconsistency, indirectness, imprecision, and publication bias. The certainty of the evidence was rated as high, moderate, low, or very low. No upgrading of certainty was applied. A Summary of Findings table was prepared using GRADEpro GDT software (version 202).

## 3. Results

### 3.1. Study Selection

**Figure 1:**
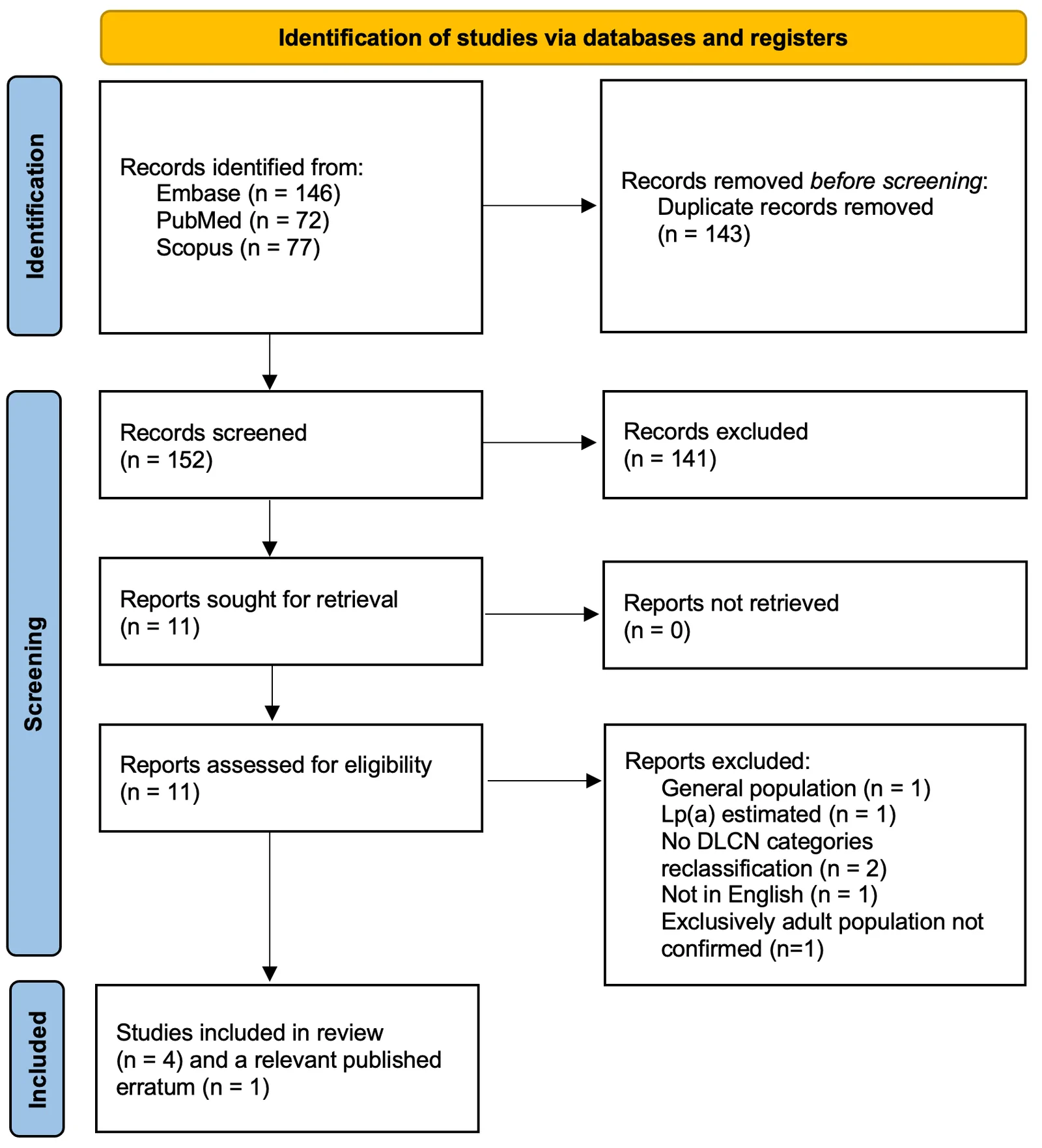
The study flowchart.

The initial database search yielded 295 records. After removal of 143 duplicates, 152 records remained and were screened based on title and abstract. In total, 141 records were excluded and 11 articles were selected for full-text assessment. Full-text articles were successfully retrieved for all records. After assessment for eligibility, 6 reports were excluded for the following reasons: (1) population not restricted to those with suspected FH [11]; (2) Lp(a) concentration estimated rather than measured [12]; (3) reclassification between DLCN diagnostic categories not reported [13,14]; (4) full text not in English [15]; (5) an exclusively adult population could not be confirmed [16]. Overall, 4 studies reported in 5 publications (including one erratum) and involving a total of 2970 individuals were included in this systematic review and meta-analysis [6,7,8,9] (Figure 1).

### 3.2. Study Characteristics

The key baseline characteristics of participants in the included studies are presented in Table 1.

The detailed study-level characteristics are provided in Table 2.

The results of the risk-of-bias and certainty assessments are presented in the Supplementary Material (Figure S1 and Table S2, respectively).

### 3.3. Downclassification of Clinical FH Diagnosis After Adjustment for Lipoprotein(a)

**Figure 2:**
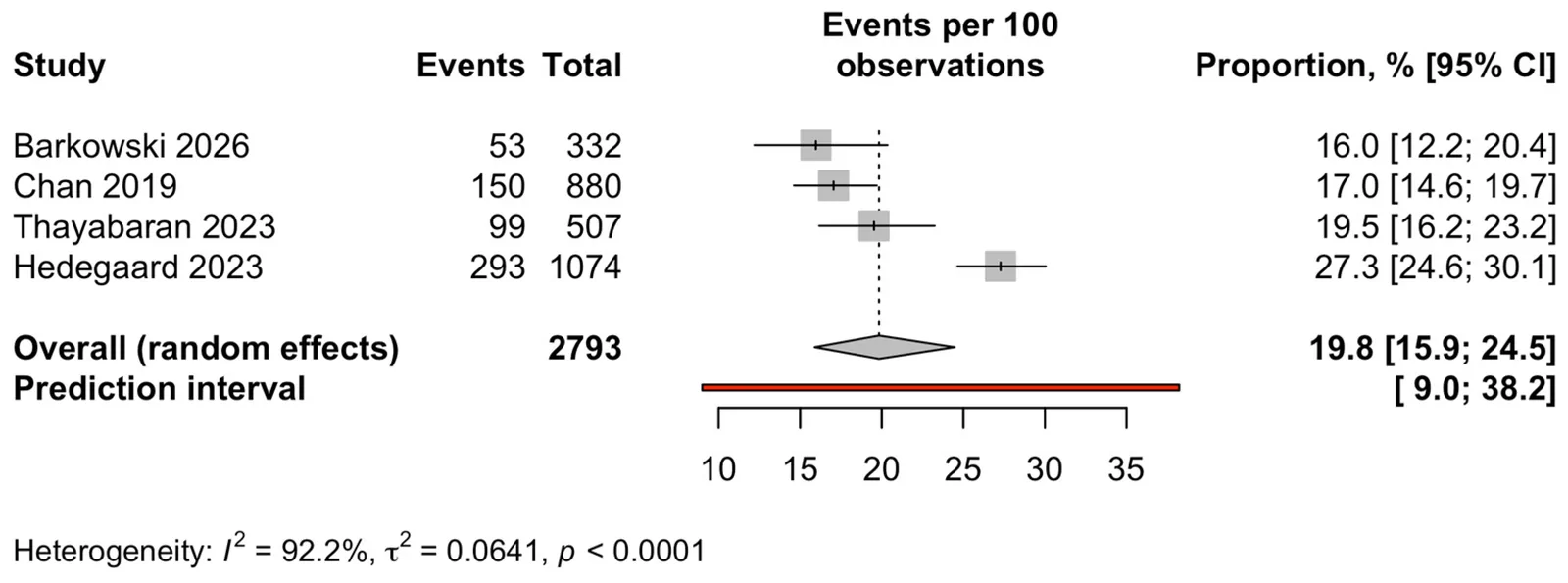
Forest plot for the primary outcome: proportion of participants whose clinical FH classification changed to a lower diagnostic category after adjustment of LDL-C for its estimated Lp(a)-cholesterol content [6,7,8,9].

Four studies included a total of 2793 individuals with possible, probable, or definite DLCN FH classification at baseline. Pooled analysis showed that 19.8% (95% CI, 15.9–24.5) of these individuals were reclassified to a lower diagnostic category after adjustment of LDL-C for its estimated Lp(a)-cholesterol content (Figure 2). The 95% prediction interval was 9.0–38.2%. Between-study heterogeneity was substantial (I^2^ = 92.2%; *p* < 0.0001).

Leave-one-out sensitivity analyses yielded pooled proportions ranging from 17.6% to 21.1% (Supplementary Material, Figure S2).

### 3.4. Reclassification from Possible to Unlikely FH After Adjustment for Lipoprotein(a)

**Figure 3:**
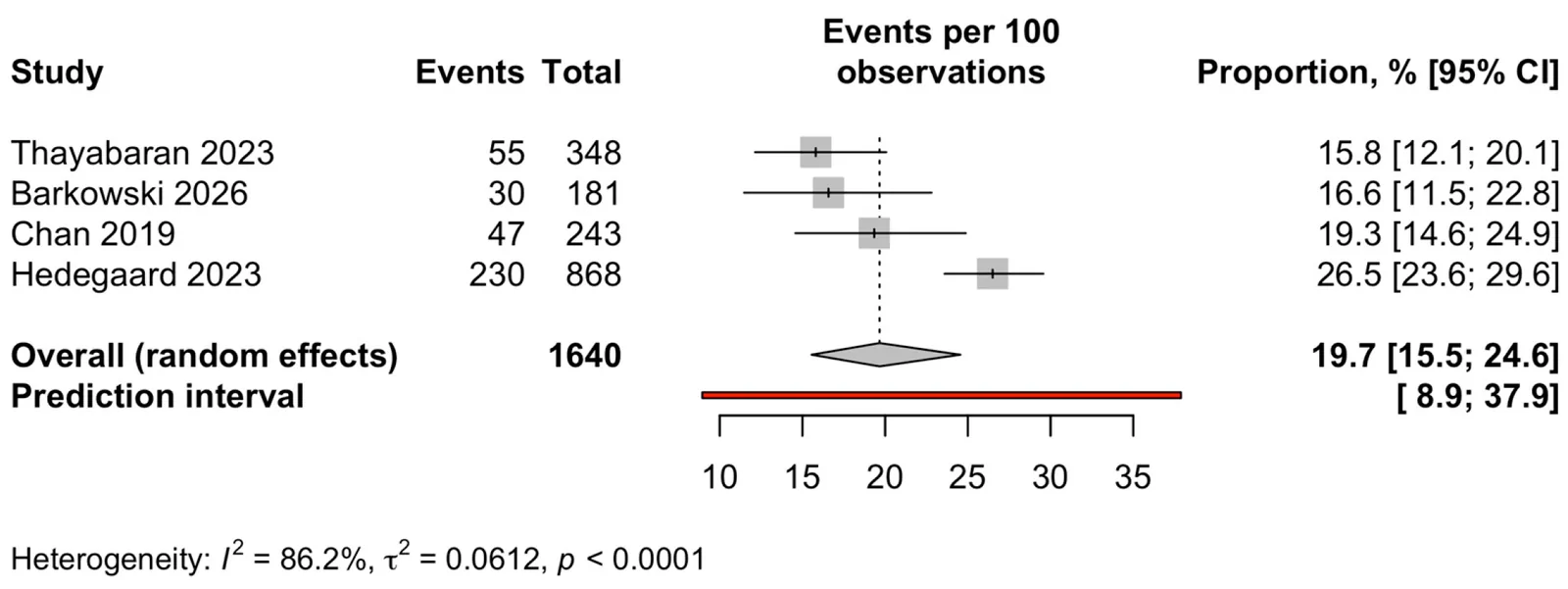
Forest plot for the secondary outcome: proportion of participants reclassified from possible to unlikely FH after adjustment of LDL-C for its estimated Lp(a)-cholesterol content [6,7,8,9].

Four studies included a total of 1640 individuals with possible DLCN FH classification at baseline. Pooled analysis showed that 19.7% (95% CI, 15.5–24.6) of these individuals were reclassified to unlikely FH after adjustment of LDL-C for its estimated Lp(a)-cholesterol content (Figure 3). The 95% prediction interval was 8.9–37.9%. Between-study heterogeneity was substantial (I^2^ = 86.2%; *p* < 0.0001).

Leave-one-out sensitivity analyses yielded pooled proportions ranging from 17.1% to 21.3% (Supplementary Material, Figure S3).

### 3.5. Other Outcomes

Changes in LDL-C concentration and clinical FH diagnostic score were summarized descriptively because data on paired changes and their variability were insufficient for meta-analysis. The included studies reported mean or median LDL-C concentrations both at baseline and after adjustment for estimated Lp(a)-cholesterol (Table 3).

Barkowski et al. also reported that the mean DLCN score changed from 5.4 (SD, 3.3) to 5.0 (SD, 3.4) after adjusting LDL-C for Lp(a) cholesterol [6].

Changes in the diagnostic performance of the DLCN score for identifying genetically confirmed FH after LDL-C adjustment for estimated Lp(a)-cholesterol are presented in Table 4.

## 4. Discussion

The main findings of our study are: (1) about one in five patients with possible, probable, or definite FH according to the DLCN score is reclassified to a lower diagnostic category after LDL-C adjustment for Lp(a)-cholesterol; (2) one in five patients with possible FH according to the DLCN score is reclassified to the unlikely FH category after adjustment for Lp(a)-cholesterol.

Our study provides quantitative pooled estimates for important clinical questions. The pooled proportions remained broadly similar in sensitivity analyses. Of note, omitting the study by Hedegaard et al. yielded largely similar pooled proportions while substantially reducing heterogeneity (I^2^ from 92.2% to 4.5% for the primary endpoint and from 86.2% to 0% for the secondary endpoint). This was observed for both the primary and secondary outcomes. Potential explanations for the observed heterogeneity include differences in the baseline distribution of the DLCN categories. In Hedegaard et al., 74% of the cohort had “possible” FH and only 5% had definite FH. Patients with “possible” FH (scores 3–5) may be more susceptible to downward reclassification. Other potentially important contributors include differences in Lp(a) assay type and units across studies, as well as differences in the proportion of patients on lipid-lowering therapy.

Some included studies reported improved discrimination for genetically confirmed FH after correction, but diagnostic performance was not pooled. However, it is important to note that the primary endpoint comprises all downward reclassifications, including definite to probable and probable to possible. Therefore, downward reclassification does not necessarily exclude FH. The secondary outcome refers to the possible-to-unlikely transition and therefore estimates the proportion of patients with possible FH at baseline who move to the unlikely category after adjustment for Lp(a)-cholesterol.

Importantly, potential reclassification of the clinical FH category must be interpreted in the context of its diagnostic performance. Although the number of false-positive classifications may decrease, the risk of missing patients with genetically confirmed FH may increase. As reported by Thayabaran et al., in their diagnostic-accuracy sample of 608 participants using a DLCN threshold of ≥3 points and the 30% correction, the increase in specificity from 18.2% to 28.7% was accompanied by a decrease in sensitivity from 90.0% to 86.7% [9]. Therefore, any potential reduction in genetic testing should be weighed against the risk of missed FH diagnoses. Nevertheless, whether the risk of missing mutation carriers outweighs the healthcare cost savings of reducing unnecessary genetic testing warrants further investigation. Of note, the 2022 European Atherosclerosis Society consensus statement does not recommend routine correction of LDL-C for Lp(a)-cholesterol but lists patients with clinically suspected FH as a potential exception [4].

Of note, the contribution of cholesterol to total Lp(a) mass has been reported to range from 5.8% to 57.3% between individuals [17]. Two of the included studies evaluated several correction factors: 17.3%, 30.0% and 45.0%. In this study, we selected the conventional 30.0% correction, as specified in the protocol.

Crucially, a lower DLCN category does not necessarily mean lower overall cardiovascular risk and should not constitute the basis for reducing preventive measures. High Lp(a) levels are associated with increased cardiovascular risk [4].

Several limitations need to be acknowledged. First, only four studies were included, limiting the evidence base. The small number of studies makes between-study variance estimation highly uncertain, and the resulting wide 95% prediction intervals should therefore be interpreted with caution. The pooled estimates should be interpreted as averages across selected referral populations with limited generalizability. Second, different Lp(a) assays were used, and some cohorts required conversions from molar to mass units. Third, this meta-analysis was restricted to the DLCN criteria. Therefore, these findings cannot be automatically extrapolated to Simon Broome or other clinical diagnostic criteria for FH. Fourth, the use of a fixed 30% correction factor may affect the pooled reclassification estimates, given the high interindividual variability in the cholesterol content of Lp(a). Nevertheless, our study has several strengths. First, all included studies evaluated adults referred for suspected FH. Second, the same diagnostic criteria and correction factor were used in all included studies. Third, the sensitivity analyses showed broadly similar pooled proportions.

To conclude, in the selected referral populations, correction of LDL-C for estimated Lp(a)-cholesterol resulted in downward DLCN reclassification in approximately one in five adults with at least possible FH. One in five adults with possible FH was reclassified to unlikely FH after adjustment. The clinical utility of this adjustment, including its consequences for genetic testing and missed FH diagnoses, needs to be investigated.

## Figures and Tables

**Table 1:** Baseline characteristics of participants in the included studies. Continuous variables are presented as mean (standard deviation) for Barkowski et al. and Chan et al., as median [10th–90th percentiles] for Hedegaard et al. and as median [interquartile range] for Thayabaran et al. Categorical variables are presented as counts, counts/denominators, or percentages.

	Barkowski 2026 [6]	Chan 2019 [7]	Hedegaard 2023 [8]	Thayabaran 2023 [9]
Age [years]	54.8 (13.9)	49.3 (13.1)	52.7 [30.2–67.9]	49 [40–57]
Female sex [%]	61.7%	56.1%	52.4%	57.1%
LDL-C before Lp(a) correction	267 (95.9) mg/dL	254 (67) mg/dL	213 [189–267] mg/dL ^1^	228 [209–255] mg/dL ^1^
Lp(a) [mg/dL]	45.5 (76.9)	n/d ^2^	15 [7–110]	28.8 [10.8–75.5]
Lipid-lowering treatment [%]	n/d	Approximately 25%	27.8%	11.9%
FH genetically confirmed	152/384	283/907	155/700 ^3^	108/513
DLCN categories				
Unlikely	52	27	92	6
Possible	181	243	868	348
Probable	84	354	147	104
Definite	67	283	59	55

^1^ Converted from mmol/L to mg/dL by multiplying by 38.67. ^2^ “n/d” refers to the absence of a reported mean or median Lp(a) concentration for the overall cohort. ^3^ Genetic testing was performed in only a subset of patients rather than the entire cohort. Abbreviations: DLCN, Dutch Lipid Clinic Network; FH, familial hypercholesterolemia; LDL-C, low-density lipoprotein cholesterol; Lp(a), lipoprotein(a); n/d, no data.

**Table 2:** Study-level characteristics of the included studies.

	Barkowski 2026 [6]	Chan 2019 [7]	Hedegaard 2023 [8]	Thayabaran 2023 [9]
Number of participants	384	907	1166	513
Country of origin	Germany	Australia	Denmark	United Kingdom
Enrollment period	2010–2023	n/d	2020–2021	n/d
Lp(a) measurement original unit	mg/dL	g/L	mg/dL or nmol/L	mg/L or nmol/L
Lp(a) conversion between molar and mass units	n/a	n/a	Lp(a) in nmol/L = 2.18 multiplied by Lp(a) in mg/dL minus 3.83	assay-specific conversion factor of 5 mg/nmol
Lp(a) assay	n/d	Quantia Lp(a) assay	3 different laboratory-based assays from Denka	IMMAGE assay (mg/L) and Randox assay (nmol/L)
LDL-C measurement	n/d	estimated by the Friedewald equation; in 4 patients with triglyceride levels >400 mg/dL, measured by direct assay	estimated by the Friedewald equation; in a few instances by direct measurement	estimated by the Friedewald equation
LDL-C in patients on lipid-lowering treatment	calculated using a respective drug-specific correction factor	adjusted for statin therapy in individuals receiving therapy	corrected for the lipid-lowering treatment	n/d

Abbreviations: LDL-C, low-density lipoprotein cholesterol; Lp(a), lipoprotein(a); n/a, not applicable; n/d, no data.

**Table 3:** LDL-C concentrations before and after adjustment for Lp(a) cholesterol. LDL-C was corrected for estimated Lp(a)-cholesterol, calculated as 30% of Lp(a) mass. Corrected LDL-C (mg/dL) = uncorrected LDL-C (mg/dL) − 0.30 × Lp(a) (mg/dL). Results based on this correction were used in all four studies. Continuous variables are presented as mean (standard deviation) for Barkowski et al. and Chan et al., as median [10th–90th percentiles] for Hedegaard et al. and as median [interquartile range] for Thayabaran et al.

	Barkowski 2026 [6]	Chan 2019 [7]	Hedegaard 2023 [8]	Thayabaran 2023 [9]
LDL-C uncorrected	267 (95.9) mg/dL	254 (67) mg/dL	213 [189–267] mg/dL ^1^	228 [209–255] mg/dL ^1^
LDL-C corrected	253.3 (99.8) mg/dL	238 (69) mg/dL	205 [170–255] mg/dL ^1^	213 [193–240] mg/dL ^1^

^1^ Converted from mmol/L to mg/dL by multiplying by 38.67.

**Table 4:** Changes in the diagnostic performance of the DLCN score for identifying genetically confirmed FH after LDL-C adjustment for estimated Lp(a)-cholesterol. For mutation carriers newly classified as unlikely FH, N denotes the total number of mutation carriers in the main study cohort. For sensitivity and specificity assessment, a DLCN score threshold of ≥3 points was used. AUC was based on the range of clinical DLCN scores in Barkowski et al. and on dichotomized DLCN classification in Chan et al. For Chan et al., the diagnostic accuracy measures were extracted from the published correction, including the adjustment for a 30% overestimation by the Quantia Lp(a) assay. For Chan et al., the number of mutation carriers newly classified as unlikely FH was derived from Table 1 and Table 2 of the original publication and refers to all 283 mutation carriers in the full cohort; the diagnostic accuracy measures refer to the subgroup of 330 patients with Lp(a) > 50 mg/dL. For Thayabaran et al., the reclassification count refers to the main cohort of 513 patients, whereas sensitivity and specificity were assessed in an expanded cohort of 608 patients.

		Barkowski 2026 [6]	Chan 2019 [7]	Hedegaard 2023 [8]	Thayabaran 2023 [9]
Mutation carriers newly classified as unlikely FH, n/N (%)		1/152 (0.7%)	0/283 (0.0%)	n/d	4/108 (3.7%)
AUC (95% CI)	before	0.782 (0.734–0.831)	0.521 (0.466–0.576)	n/d	n/d
	after	0.803 (0.757–0.848)	0.584 (0.520–0.648)	n/d	n/d
Sensitivity	before	94%	100%	n/d	90.0%
	after	93%	100%	n/d	86.7%
Specificity	before	19%	4.2%	n/d	18.2%
	after	31%	16.8%	n/d	28.7%

## Data Availability

The data underlying this article will be shared on request to the corresponding author.

## Supplementary Materials

The following supporting information can be downloaded at https://www.mdpi.com/article/10.3390/jcm15218614/s1, Figure S1: traffic light plot of the risk-of-bias assessment; Figure S2: leave-one-out sensitivity analysis for the primary outcome; Figure S3: leave-one-out sensitivity analysis for the secondary outcome; Method S1: full search strategy used for all databases; Table S1: PRISMA 2020 Checklist; Table S2: summary of findings and certainty of evidence.

## Registration and Protocol

This systematic review was registered with the International Prospective Register of Systematic Reviews (PROSPERO) under registration number CRD420261534815. No separate protocol was prepared; the PROSPERO record served as the study protocol. Any amendments were documented when the PROSPERO record was updated.

## Abbreviations

The following abbreviations are used in this manuscript:

DLCN	Dutch Lipid Clinic Network
FH	Familial hypercholesterolemia
LDL-C	Low-density lipoprotein cholesterol
Lp(a)	Lipoprotein(a)
