# Supplementary material for: Reclassification of Clinical Familial Hypercholesterolemia Diagnoses After Adjustment of LDL-C for Lipoprotein(a) Cholesterol: A Systematic Review and Meta-Analysis
Source: J Clin Med. 2026 Nov 5;15(21):8614. doi: 10.3390/jcm15218614 (PMC13764630; doi:10.3390/jcm15218614)

SUPPLEMENTARY MATERIAL

Title:

Reclassification of clinical familial hypercholesterolemia diagnoses after adjustment of LDL-C for lipoprotein(a) cholesterol: a systematic review and meta-analysis

Names of the authors and affiliations:

Grzegorz Procyk, Adam Sukiennik, Jakub Michal Zimodro

First Department of Cardiology, Medical University of Warsaw, Warsaw, Poland

Details of the corresponding author:

Jakub Michal Zimodro, MD

jakub.zimodro@wum.edu.pl

First Department of Cardiology

Medical University of Warsaw

Banacha 1A, 02-097 Warsaw, Poland

Tel: +48 22 599-19-58

Fax: +48 22 599-19-57

Conflict of interest statement: none declared

TABLE OF CONTENTS:

Supplemental Method S1. Full search strategy used for all databases. 3

Supplemental Table S1. PRISMA 2020 Checklist. 4

Supplemental Table S2. Summary of findings and certainty of evidence. 6

Supplemental Figure S1. Traffic light plot of the risk-of-bias assessment 7

Supplemental Figure S2. Leave-one-out sensitivity analysis for the primary outcome 8

Supplemental Figure S3. Leave-one-out sensitivity analysis for the secondary outcome....... 9

Supplemental Method S1. Full search strategy used for all databases.

Embase:

('familial hypercholesterolemia':ti,ab OR 'familial hypercholesterolaemia':ti,ab) AND ('lipoprotein(a)':ti,ab OR 'lipoprotein a':ti,ab OR 'lp(a)':ti,ab) AND ('ldl-c':ti,ab OR 'ldl cholesterol':ti,ab OR 'low-density lipoprotein cholesterol':ti,ab) AND (adjust*:ti,ab OR correct*:ti,ab OR reclassif*:ti,ab OR misclassif*:ti,ab OR classif*:ti,ab)

PubMed:

("familial hypercholesterolemia"[tiab] OR "familial hypercholesterolaemia"[tiab]) AND ("lipoprotein(a)"[tiab] OR "lipoprotein a"[tiab] OR "Lp(a)"[tiab]) AND (LDL-C[tiab] OR "LDL cholesterol"[tiab] OR "low-density lipoprotein cholesterol"[tiab]) AND (adjust*[tiab] OR correct*[tiab] OR reclassif*[tiab] OR misclassif*[tiab] OR classif*[tiab])

Scopus:

TITLE-ABS("familial hypercholesterolemia" OR "familial hypercholesterolaemia") AND TITLE-ABS("lipoprotein(a)" OR "lipoprotein a" OR "Lp(a)") AND TITLE-ABS("LDL-C" OR "LDL cholesterol" OR "low-density lipoprotein cholesterol") AND TITLE-ABS(adjust* OR correct* OR reclassif* OR misclassif* OR classif*)

Supplemental Table S1. PRISMA 2020 Checklist.

Section and Topic	Item #	Checklist item	Location where item is reported
**TITLE**			
Title	1	Identify the report as a systematic review.	Title
**ABSTRACT**			
Abstract	2	See the PRISMA 2020 for Abstracts checklist.	Abstract
**INTRODUCTION**			
Rationale	3	Describe the rationale for the review in the context of existing knowledge.	Introduction
Objectives	4	Provide an explicit statement of the objective(s) or question(s) the review addresses.	Introduction
**METHODS**			
Eligibility criteria	5	Specify the inclusion and exclusion criteria for the review and how studies were grouped for the syntheses.	Methods
Information sources	6	Specify all databases, registers, websites, organisations, reference lists and other sources searched or consulted to identify studies. Specify the date when each source was last searched or consulted.	Methods
Search strategy	7	Present the full search strategies for all databases, registers and websites, including any filters and limits used.	Methods, Supplementary Method S1
Selection process	8	Specify the methods used to decide whether a study met the inclusion criteria of the review, including how many reviewers screened each record and each report retrieved, whether they worked independently, and if applicable, details of automation tools used in the process.	Methods
Data collection process	9	Specify the methods used to collect data from reports, including how many reviewers collected data from each report, whether they worked independently, any processes for obtaining or confirming data from study investigators, and if applicable, details of automation tools used in the process.	Methods
Data items	10a	List and define all outcomes for which data were sought. Specify whether all results that were compatible with each outcome domain in each study were sought (e.g. for all measures, time points, analyses), and if not, the methods used to decide which results to collect.	Methods
	10b	List and define all other variables for which data were sought (e.g. participant and intervention characteristics, funding sources). Describe any assumptions made about any missing or unclear information.	Methods
Study risk of bias assessment	11	Specify the methods used to assess risk of bias in the included studies, including details of the tool(s) used, how many reviewers assessed each study and whether they worked independently, and if applicable, details of automation tools used in the process.	Methods
Effect measures	12	Specify for each outcome the effect measure(s) (e.g. risk ratio, mean difference) used in the synthesis or presentation of results.	Methods
Synthesis methods	13a	Describe the processes used to decide which studies were eligible for each synthesis (e.g. tabulating the study intervention characteristics and comparing against the planned groups for each synthesis (item #5)).	Methods
	13b	Describe any methods required to prepare the data for presentation or synthesis, such as handling of missing summary statistics, or data conversions.	Methods
	13c	Describe any methods used to tabulate or visually display results of individual studies and syntheses.	Methods
	13d	Describe any methods used to synthesize results and provide a rationale for the choice(s). If meta-analysis was performed, describe the model(s), method(s) to identify the presence and extent of statistical heterogeneity, and software package(s) used.	Methods
	13e	Describe any methods used to explore possible causes of heterogeneity among study results (e.g. subgroup analysis, meta-regression).	Methods
	13f	Describe any sensitivity analyses conducted to assess robustness of the synthesized results.	Methods
Reporting bias assessment	14	Describe any methods used to assess risk of bias due to missing results in a synthesis (arising from reporting biases).	Methods
Certainty assessment	15	Describe any methods used to assess certainty (or confidence) in the body of evidence for an outcome.	Methods

Section and Topic	Item #	Checklist item	Location where item is reported
**RESULTS**			
Study selection	16a	Describe the results of the search and selection process, from the number of records identified in the search to the number of studies included in the review, ideally using a flow diagram.	Results, Figure 1
	16b	Cite studies that might appear to meet the inclusion criteria, but which were excluded, and explain why they were excluded.	Results
Study characteristics	17	Cite each included study and present its characteristics.	Results, Tables 1 and 2
Risk of bias in studies	18	Present assessments of risk of bias for each included study.	Supplemental Figure S1
Results of individual studies	19	For all outcomes, present, for each study: (a) summary statistics for each group (where appropriate) and (b) an effect estimate and its precision (e.g. confidence/credible interval), ideally using structured tables or plots.	Figures 2-3, Tables 3-4
Results of syntheses	20a	For each synthesis, briefly summarise the characteristics and risk of bias among contributing studies.	Results
	20b	Present results of all statistical syntheses conducted. If meta-analysis was done, present for each the summary estimate and its precision (e.g. confidence/credible interval) and measures of statistical heterogeneity. If comparing groups, describe the direction of the effect.	Results, Figures 2-3
	20c	Present results of all investigations of possible causes of heterogeneity among study results.	Supplemental Figures S2 and S3
	20d	Present results of all sensitivity analyses conducted to assess the robustness of the synthesized results.	Supplemental Figures S2, S3
Reporting biases	21	Present assessments of risk of bias due to missing results (arising from reporting biases) for each synthesis assessed.	n/a
Certainty of evidence	22	Present assessments of certainty (or confidence) in the body of evidence for each outcome assessed.	Supplemental Table S2
**DISCUSSION**			
Discussion	23a	Provide a general interpretation of the results in the context of other evidence.	Discussion
	23b	Discuss any limitations of the evidence included in the review.	Discussion
	23c	Discuss any limitations of the review processes used.	Discussion
	23d	Discuss implications of the results for practice, policy, and future research.	Discussion
**OTHER INFORMATION**			
Registration and protocol	24a	Provide registration information for the review, including register name and registration number, or state that the review was not registered.	Registration and Protocol
	24b	Indicate where the review protocol can be accessed, or state that a protocol was not prepared.	Registration and Protocol
	24c	Describe and explain any amendments to information provided at registration or in the protocol.	Registration and Protocol
Support	25	Describe sources of financial or non-financial support for the review, and the role of the funders or sponsors in the review.	Funding
Competing interests	26	Declare any competing interests of review authors.	Conflicts of Interest
Availability of data, code and other materials	27	Report which of the following are publicly available and where they can be found: template data collection forms; data extracted from included studies; data used for all analyses; analytic code; any other materials used in the review.	Data Availability Statement

From: Page MJ, McKenzie JE, Bossuyt PM, Boutron I, Hoffmann TC, Mulrow CD, et al. The PRISMA 2020 statement: an updated guideline for reporting systematic reviews. BMJ 2021;372:n71. doi: 10.1136/bmj.n71.
This work is licensed under CC BY 4.0. To view a copy of this license, visit https://creativecommons.org/licenses/by/4.0/

Supplemental Table S2. Summary of findings and certainty of evidence.

No. of studies	Certainty assessment						Effect			Certainty	Importance
	Study design	Risk of bias	Inconsistency	Indirectness	Imprecision	Other considerations	Number of events	Number of individuals	Rate (95% CI)		
**FH downward reclassification from possible, probable or definite at baseline**											
4	non-randomized studies	serious	serious	not serious	not serious	none	595	2793	event rate 19.8 per 100 (15.9 to 24.5)	⊕⊕◯◯ Low	CRITICAL
**FH downward reclassification from possible to unlikely**											
4	non-randomized studies	serious	serious	not serious	not serious	none	362	1640	event rate 19.7 per 100 (15.5 to 24.6)	⊕⊕◯◯ Low	CRITICAL

Supplemental Figure S1. Traffic light plot of the risk-of-bias assessment across the included studies.

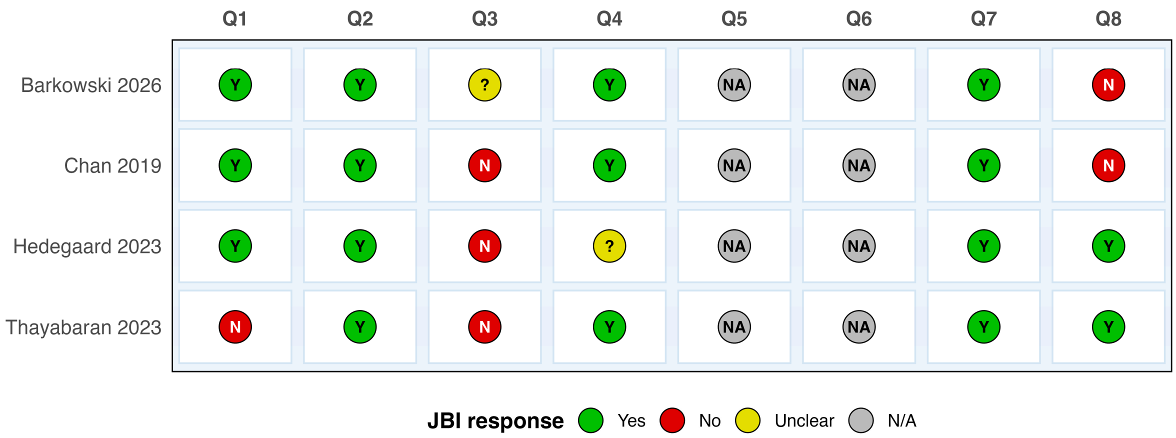

Q1 = Were the criteria for inclusion in the sample clearly defined?
Q2 = Were objective, standard criteria used for measurement of the condition?
Q3 = Was the exposure measured in a valid and reliable way?
Q4 = Were the outcomes measured in a valid and reliable way?
Q5 = Were confounding factors identified?
Q6 = Were strategies to deal with confounding factors stated?
Q7 = Was appropriate statistical analysis used?
Q8 = Were the study subjects and the setting described in detail?

Supplemental Figure S2. Leave-one-out sensitivity analysis for the primary outcome: proportion of participants whose clinical FH classification changed to a lower diagnostic category after adjustment of LDL-C for its estimated Lp(a)-cholesterol content.

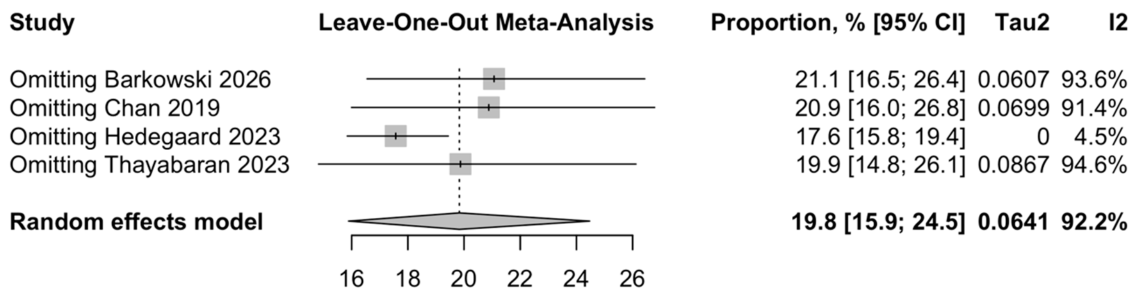

Supplemental Figure S3. Leave-one-out sensitivity analysis for the secondary outcome: proportion of participants reclassified from possible to unlikely FH after adjustment of LDL-C for its estimated Lp(a)-cholesterol content.

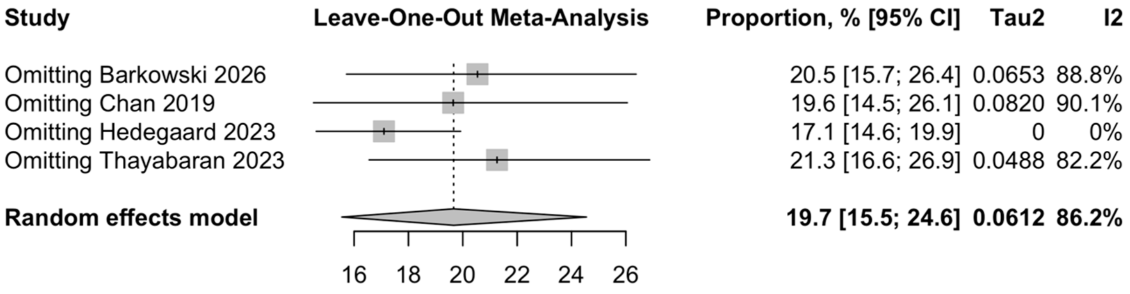

Supplement: Supplementary file 1 [file jcm-15-08614-s001.zip › jcm-4661427-supplementary.pdf]
